# Factors associated with pregnancy rate in fixed-time embryo transfer in cattle under humid-tropical conditions of México

**DOI:** 10.1590/1984-3143-AR2020-0007

**Published:** 2020-06-26

**Authors:** Alfonso Pérez-Mora, José Candelario Segura-Correa, Jorge Alonso Peralta-Torres

**Affiliations:** 1 Laboratorio ReproSur, Villahermosa, Tabasco, México; 2 Campus de Ciencias Biológicas y Agropecuarias, Universidad Autónoma de Yucatán, Mérida, Yucatán, México; 3 División Académica de Ciencias Agropecuarias, Universidad Juárez Autónoma de Tabasco, Villahermosa, Tabasco, México

**Keywords:** body condition, embryo *in vivo* and *in vitro*, female type, race, recipient synchronization protocol

## Abstract

The objective was to determine the effect of some factors on pregnancy rate of fixed-time embryo transfer (FTET), in cows and heifers kept under Mexican tropical conditions. Recipients females (n=405) grazing in pastures were selected according to breed group (Zebu and crosses), parity (nulliparous and multiparous), body condition score (BCS) and the presence of a corpus luteum (CL). The females were synchronized on day 0 using a progesterone vaginal device and 2 mg estradiol benzoate (EB), two groups were established. Group 1 (conventional protocol) were animals in which the progesterone device was removed on day 7. At this time, also received an injection of 50 mg cloprostenol sodium and 1 mg estradiol cypionate. Animals also received 300 IU (heifers) or 360 IU (cows) of eCG. Group 2 (J-Synch protocol) were animals in which the progesterone device was removed on day 6. Cloprotenol and eCG injections were applied as in Group 1. Additionally, on day 9, animals of group 2 received 0.01 mg buserelin acetate. Embryo transfer of *in vivo* or *in vitro* was done on day 16 and pregnancy diagnosis was realized by ultrasonography on days 23 and 53 after FTET. Statistical analyses were carried out using Chi-square tests and logistic regression. Pregnancy rate varied between farms (P<0.05). The highest pregnancy rate was for multiparous cows (66%). The recipient utilization rate was better in the J-Synch protocol (85%), and *in vivo* embryos (75%) had higher pregnancy rate. The diameter of the follicle and the CL had no effect on pregnancy rate (P>0.05). However, the logistic regression determined that the only significant factor on pregnancy rate was the type of embryo. In conclusion, pregnancy rate in FTET females was higher for *in vivo* embryos than for *in vitro* embryos in cows evaluated under humid tropical conditions in Mexico.

## Introduction

In Mexico, as in other parts of the world, the implementation of reproductive biotechnologies such as multiple ovulation and embryo transfer (MOET), and *in vitro* fertilization (IVF) are important tools for disseminating animals with high genetic merit, to improve productive performance of cattle herds. However, the success of embryo transfer chiefly depends on factors associated with recipient selection, synchronization protocol used and embryo production method. Some factors to consider when selecting female recipients are type of cattle, *Bos indicus*, *Bos taurus* or the crosses of both (McEvoy et al., 2006), parity (nulliparous or multiparous), body condition score (BCS), management, reproductive and nutritional status (Bó et al., 2012; Wu and Zan, 2012). In relation to the synchronization of the recipient, protocols are applied that allow embryo transfer (ET) without estrus detection, known as fixed-time embryo transfer (FTET) protocol. The most common protocol consist in the application of 2 mg of estradiol benzoate (EB) at the moment of the device insertion, which is defined as day 0 of the protocol. Seven days after the devise is retired and prostaglandin (PGF_2α_), 1 mg de estradiol cypionate (EC) and 300 IU of equine chorionic gonadotrophin (eCG) is applied (Bó and Cedeño, 2018). The J-Synch protocol consist in the withdraw of the device at day 6, and the application of PGF_2α_ and 300 IU of eCG. Seventy-two hours later (day 9), 100 µg of buserelin acetate is applied (De la Mata and Bó, 2012). The J-Synch protocol extends the proestrus (increase the time interval between withdrawal of the device and ovulation) and improves pregnancy rate of FTET in beef heifers (Menchaca et al., 2017). The benefits of the J-Synch protocol is related to an increase in growth rate of the dominant ovulatory follicle, associated to greater pre-ovulatory estradiol and post-ovulatory progesterone concentrations. This is explained because of a complex modulation of the expression of the estrogen and progesterone receptors and other growth factor, such as insulin-like growth factor (IGF1), which prepare the uterine environment to receive the embryo and for pregnancy establishment (De la Mata and Bó, 2012; Bó et al., 2016; De la Mata et al., 2018; Macmillan et al., 2020; Núñez-Olivera et al., 2020). Finally, the method to produce the embryo (*in vivo* or *in vitro*) transferred, influences pregnancy rate (Pontes et al., 2009; Ferraz et al., 2016). Furthermore, embryonic quality and development is of vital importance (Rodrigues et al., 2018), as well as the site of placement of the embryo and the time to complete the embryo transfer (Roper et al., 2018).

Under Mexican tropical conditions, the availability of recipients is scarce; therefore, to optimize the use of embryo recipients in ET programs, a better understanding of the factors that affect the pregnancy rate is necessary. The objective of this study was to evaluate some factors that affected the success of embryo transfer in bovine recipient raised under the humid tropical conditions in Mexico.

## Methods

### Study area

The experiment was conducted in Tabasco, Mexico, located between parallels 18°39’03” and 17°15’03” north latitude and 90°59’15” and 94°07'48” west longitude, at 10 m altitude. The climate of the region is warm humid with average annual temperature and rainfall of 26 °C and 1,627 mm, respectively (INEGI, 2017).

### Selection criteria of recipients and management

The procedures were performed in accordance with the Mexican Official Standard guideline 051-ZOO-1995 and the Mexican Official Standard of technical specifications for production, care and use of experimental animals. The project was approved by the Ethics Committee of Universidad Juárez Autónoma de Tabasco (approval number: 0240).

Four hundred and five recipients were involved, the selection criteria of the recipients were:

*Breed: Bos indicus* (Zebu) and *Bos indicus* × *Bos taurus* (crosses) females.

*Female parity*: nulliparous (24 to 36 months old) and multiparous (2-5 parities) females.

*Body condition score* (BCS): Females were selected with 5 and 8 BCS points, in a scale from 1 to 9, where 1 is emaciated and 9 is obese (Ayala et al., 1995).

*Cyclicity*: Determined by the presence of a corpus luteum (CL), confirmed by ultrasonography.

The females were distributed in eight farms, and all herds enrolled had similar management and fed under rangeland conditions, with *ad libitum* access to water and mineral salt. Owners of the farms were recommended to improve the care and management of embryo recipients reducing animal science practices (vaccination, deworming, etc.), providing forage of good quality, and strategic feed supplementation.

### Synchronization protocols

#### Conventional protocol

**Figure 1:**
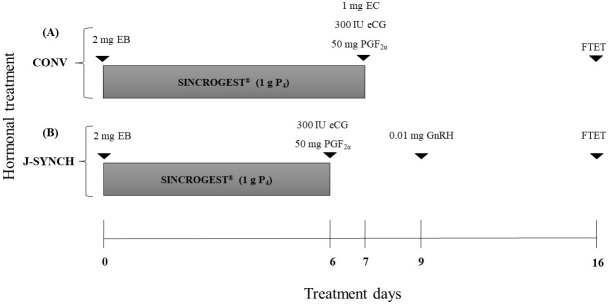
(A) Conventional and (B) J-Synch synchronization protocols used in recipients. EB (estradiol benzoate), P_4_ (progesterone), PGF_2α_ (prostaglandin F_2α_; cloprostenol sodium), eCG (equine chorionic gonadotropin), GnRH (gonadotropin releasing hormone; buserelin acetate) and FTET (fixed-time embryo transfer).

All the recipients (n = 201), on day 0, were applied a vaginal device impregnated with 1 g of progesterone (Sincrogest^®^, Ourofino), and then intramuscularly 2 mg of EB (Sincrodiol^®^, Ourofino). After removal of the device (day 7), cows were given 50 mg of sodium cloprostenol (Sincrocio^®^, Ourofino) plus 360 IU of eCG; whereas heifers were applied 300 IU of eCG (Novormon^®^, Virbac) plus 1 mg of EC (SincroCP^®^, Ourofino), intramuscularly (Figure 1A).

#### J-Synch protocol

All the recipients (n = 204), on day 0, received a vaginal device impregnated with 1 g of progesterone plus 2 mg of EB applied intramuscularly. At the withdrawal of the device (day 6), cows were given 50 mg of cloprostenol sodium plus 360 IU of eCG; whereas heifers were applied 300 IU of eCG, on day 9, plus 0.01 mg of buserelin acetate (Buserelina^®^, Zoovet, intramuscularly (De la Mata and Bó, 2012; Figure 1B).

### Embryo production

The *in vivo* and *in vitro* embryos were produced by conventional methods (Pontes et al., 2009), were arising from donor (*Bos indicus*) cows. The GENEMEX^®^ International Trading Company carried out the *in vitro* embryo production.

### Evaluation and embryo transfer

Embryos were classified under a stereoscope with 45X magnification; the viable embryos were further classified based on their developmental stage and grade of quality according to the criteria of the International Embryo Transfer Society (IETS; Stringfellow and Seidel, 1990). Embryos transferred *in vivo* were classified according to the stage of development: 4, compact morula; 5, early blastocyst; 6, blastocyst; and 7, expanded blastocyst, all quality 1 (excellent/good) and 2 (fair). *In vitro* transferred embryos were grade 7, expanded blastocyst and quality 1 (excellent/good).

Embryo transfer was performed in the ipsilateral horn to the CL, only using recipients with CL ≥ 15 mm and follicles ≤ 12 mm. Embryo transfer was done on a fixed time on day 16.

### Pregnancy diagnosis

The pregnancy was diagnosed 23 and 53 days after FTET (pregnancy time of 30 and 60 days, considering the age of the embryo) by means of a real-time ultrasound equipment (ECM, ImaGo^®^) with a 5 MHz linear transducer.

### Variables measured

*Transferred*: the total number of females selected, synchronized and embryos transferred.

*Pregnant*: total transferred females diagnosed pregnant at 30 and 60 days.

*Embryonic loss*: recipients diagnosed pregnant at 30 days, and which did not maintain pregnancy 60 days after.

*Pregnant/treated*: females diagnosed pregnant considering all synchronized females.

### Statistical analysis

To determine the effect of farm, breed, female parity, body condition score (at the start of the study), hormonal treatment and type of embryo, Chi-square or Fisher's exact tests were used. The level of significance was 5%. Subsequently, those factors with significance greater than 20% were further evaluated using logistic regression procedures that included in addition to the aforementioned factors, the diameter of the follicle and the diameter of the CL (SAS Institute Inc., 2015).

## Results

Farms differences for embryos transferred were observed for all traits evaluated (except for transferred females). The higher pregnancy rates at 30 and 60 days were observed in farms II, VI and VII with pregnancy rates at 60 days of 65%, 74% and 83%, respectively. The lowest pregnancy rate was for farm III with 25% (Table 1). Four of the farms had an embryonic loss lower than 7%.

**Table 1:** Relative frequencies (%) by farm for transferred females, pregnancy rate at 30 and 60 days, embryonic loss and pregnancy rate of treated females.

**Farms**	**N**	**Transferred (%)**	**Pregnant 30 d (%)**	**Pregnant 60 d (%)**	**Embryo loss (%)**	**Pregnant/ treated (%)**
I	59	77.97 (46/59)^a^	65.22 (30/46)^bc^	60.87 (28/46)^bc^	6. 67(2/30)^bc^	47.46 (28/59)^a^
II	26	88.46 (23/26)^a^	91.30 (21/23)^a^	65.22 (15/23)^ab^	28.57 (6/21)^a^	57.69 (15/26)^a^
III	28	85.71 (24/28)^a^	29.17 (7/24)^d^	25 (6/24)^d^	14.28 (1/7)^abc^	21.43 (6/28)^b^
IV	41	87.80 (36/41)^a^	66.67 (24/36)^bc^	55.56 (20/36)^bc^	16.67 (4/24)^ab^	48.78 (20/41)^a^
V	92	75.00 (69/92)^a^	63.77 (44/69)^bc^	57.97 (40/69)^bc^	9.09 (4/44)^abc^	43.48 (40/92)^a^
VI	61	77.05 (47/61)^a^	78.72 (37/47)^ab^	74.47 (35/47)^ab^	5.40 (2/37)^bc^	57.38 (35/61)^a^
VII	43	72.09 (31/43)^a^	83.87 (26/31)^ab^	83.87 (26/31)^a^	0 (0/26)^c^	60.46 (26/43)^a^
VIII	55	81.82 (45/55)^a^	55.56 (25/45)^c^	53.33 (24/45)^c^	4 (1/25)^bc^	43.63 (24/55)^a^
**Total**	405	79.26 (321/405)	66.67 (214/321)	60.43 (194/321)	9.34 (20/214)	47.90 (194/405)

abcd: Different letters by columns indicate significant differences (P<0.05). The following abbreviation was used: N (number).

In the Chi-square or Fisher tests, the significant factors were parity group and type of protocol for transferred females, and type of parity and type of embryo for pregnancy rate at 30 and 60 days (Table 2). No effect of any of the factors studied was found for embryonic loss and pregnant/treated females (Table 2). However, logistic regression only showed significant effect of the type of embryo on pregnancy rate. The likelihood of a cow being pregnant was greater for an *in vivo* transferred embryo that for an *in vitro* embryo (OR = 2,436; 95% CI: 1,438-4,128). In addition, the diameter of the follicle and the CL at embryo transfer had no effect on pregnancy rate (P>0.05).

**Table 2:** Relative frequencies (%) by exploratory factor for transferred females, pregnancy rate at 30 and 60 days, embryonic loss and pregnancy rate of treated females.

**Factor**	**N**	**Transferred (%)**	**P value** ^*^	**N**	**Pregnant 30 d (%)**	**P value** ^*^	**N**	**Pregnant 60 d (%)**	P value*	**N**	**Embryo loss (%)**	P value*	**N**	**Pregnant/treated (%)**	P value*
**Breed**			0.7228			0.2914			0.4516			0.6024			0.5201
Zebu	234	81.20		190	65.26		190	59.47		190	5.79		234	48.29	
Cross	153	79.74		124	70.97		124	63.71		124	7.26		153	51.63	
**Parity group**			0.0294			0.0066			0.0227			0.5099			0.4145
Nulliparous	151	86.09		132	59.09		132	53.79		132	5.30		151	47.02	
Multiparous	236	77.12		182	73.63		182	66.48		182	7.14		236	51.27	
**BCS at day 0**			0.5236			0.0854			0.1343			0.9283			0.4937
6	85	82.35		70	68.57		70	62.86		70	5.71		80	51.76	
7	230	81.74		190	63.68		190	57.37		190	6.32		230	47.39	
8	71	76.06		54	79.63		54	72.22		54	7.41		71	54.93	
**Protocol**			0.0404			0.3405			0.9206			0.0989			0.4453
Conventional	193	77.68		150	69.33		150	60.67		150	8.67		193	47.17	
J-Synch	198	84.85		168	64.29		168	60.12		168	4.17		198	51.01	
**Type of embryo**						<0.0001			0.0004			0.4065			
*In vivo*				100	83.00		100	75.00		100	8.00				
*In vitro*				216	59.72		216	54.17		216	5.56				

* P values according to Chi-square or Fisher exact tests. The following abbreviation was used: N (number).

## Discussion

To our knowledge, this is the first study addressed to analyze some factors related with the pregnancy rate of recipients submitted to ET in Mexican tropical climate conditions. The results of this study indicated that only the type of embryo affected pregnancy rate. However, other factors also affected the pregnancy rate of FTET.

The lack of significant difference for pregnancy rate of FTET between the two protocols here evaluated disagree from Núñez-Olivera et al. (2020) and Menchaca et al. (2017) results, who observed a greater pregnancy rate for the J-Synch protocol using IAFT and FTET, respectively. Here, a higher utilization rate of recipients with the J-Synch protocol (85%) was found and a trend (P=0.0989) to lower embryonic loss, compared to the conventional protocol. This strengthen the theory that J-Synch fosters long-lasting proestrus of a more receptive (embryotrophic) uterine environment, greater serum concentrations of progesterone and larger size of CL, which cause minor embryo losses (Gonella et al., 2010; Diskin et al., 2016; Binelli et al., 2017; De la Mata et al., 2018). Therefore, the results here found favor the use of J-Synch protocol under the management and tropical conditions of Mexico.

The highest pregnancy rate for embryos transferred *in vivo* at 30 and 60 days (83% and 75%, respectively) found in this study differs considerably from others. The few studies in Mexico, which evaluate *in vivo* and *in vitro* embryo production in *Bos indicus* cattle report pregnancy rates lower than 45% (Alarcón et al., 2010; Lozano-Domínguez et al., 2010). There are several differences between embryos generated *in vivo* and *in vitro* (Hansen and Block, 2004). Based on the current findings, the lack of differences in pregnancy rate of embryos transferred is very important to identify donors that respond better to *in vivo* or *in vitro* embryos transferred. Both biotechnologies count with advantages and disadvantages; therefore, they could be used in an alternate way, to get the mayor number of progeny from animals of high genetic merit.

The highest rate of transferred recipient females was observed for the nulliparous group (86%); however, the highest pregnancy rate was for the multiparous cows (73.6% and 66.5% at 30 and 60 days, respectively). This pregnancy rate is similar to that reported by Looney et al. (2006), who notified a higher pregnancy rate in multiparous (66%) compared to nulliparous (43%) females. However, other authors (Hasler, 2001; Ferraz et al., 2016) reported higher pregnancy rates in nulliparous females (79 and 42%) compared to the multiparous cows (61 and 31.6%). Therefore, parity and associated management of the females could be an important factor for a better pregnancy rate.

The significant difference between farms in pregnancy rate, agree with Stroud and Hasler (2006) results, who report that pregnancy rates of *in vivo* embryos transferred is associated with poor management in the farm. In addition, the high pregnancy observed in more farms is probably associated to the fact that only excellent quality embryos were transferred, a strict selection of recipients was carried out, management criteria were established, such as no excessive management of females short after ET, and they were given forage of good quality and quantity, and if possible were feed supplemented. The poor performance, here found, in farm III, it is possibly due to the fact that animals in such farm were managed in excess (vaccinated, dewormed etc.) and were not given adequate feeding. It is known that excessive management of recipients is one of the main causes of low pregnancy rate after FTET. Farm effect is expected in most studies of reproductive traits, because it is a complex factor difficult to control. The effects could be associated to many variables such as micro-region climate, management and infrastructure differences, as well as system of production and nutritional aspects.

The lack of difference, here found, between Zebu and crossbred females recipients for pregnancy rate, was probably due the fact that *in vivo* e *in vitro* embryos were obtained from Zebu donors. However, Marinho et al. (2015) in crossbred recipients, Gyr and Holstein cow donors obtained pregnancy rates of 46.1% in Gyr and 32.5% in Holstein cows. The better results of Gyr cows could be due to the hardiness of *Bos indicus* because they are more resistant to high temperatures and high humidity. In this sense, Gjørret et al. (2003) indicated that in Holstein embryos, higher incidence of apoptotic cells was observed in blastocysts derived *in vitro* than in the *in vivo* counterpart.

There were no differences in pregnancy rate of female recipients with different BCS at the beginning of synchronization (day 0). These results differ from those by Mapletoft et al. (1986), who found better pregnancy rates in recipients with body condition (scale 1-5) of 3 (55%) and 2 (53%) compared with recipients with BCS of 1 or lower (44%). This could be explained because, in most of the farms here studied, females that were in not good body condition at the beginning of the embryo transfer program, were given supplementary feed to improve their BCS and get into a positive energetic balance. It is known, that nutrition is a key factor in all aspects of reproduction and it is especially critical for recipient females. BCS and feed energy are major factors regulating reproductive successes and/or failures in recipients. It is difficult to expect superior results in donors or recipients with nutritional deficiencies (Looney et al., 2006). In this sense, it is very important for female recipients not to be on negative energy balance, especially during the first 45 days after FTET.

In this study, the size of the CL had no effect on the pregnancy rate of FTET. This finding differs from those of Baruselli et al. (2003) and Nasser et al. (2009), who observed that as the diameter of the CL increases, so do the pregnancy rate. It is well established that larger CL secrete more P_4_ and this may have a positive effect on pregnancy recognition and consequently pregnancy rates in embryo transferred programs (Baruselli et al., 2010). In addition, the diameter of the follicle in ET (first follicular wave) had no effect on pregnancy rate. This is probably because the size of the follicle is not related to the late luteal phase that prematurely increases estrogen receptor-α abundance and exacerbates PGF release (Cerri et al., 2011).

Finally, in this study, females with reproductive merit were selected to be recipients and only the factors evaluated were the variants in the FTET program. The good pregnancy rate found in the present study are probably due to the quality of the recipients (cycling, good BCS, CL ≥ 15 mm and follicles ≤ 12 mm) and the quality of the transferred embryos (quality 1 and 2). Baruselli et al. (2011) indicated that progesterone-synchronized recipients usually exceed 50% of pregnant females, when both embryos and recipients are of high quality. Other authors have reported up to 78.8 and 56.8% of pregnant females with *in vivo* and *in vitro* embryos, respectively (Hasler, 2001; Chebel et al., 2008). Therefore, it is important to emphasize that *Bos indicus* and their crosses could be use in FTET programs, under environmental conditions prevailing in the humid tropics. However, more research under tropical conditions are needed before high pregnancy rates are common in most farms.

## Conclusion

Of the female factors here evaluated, only the type of embryo (*in vivo*, *in vitro*) was associated with a higher pregnancy rate in cows evaluated under humid tropic conditions in Mexico. In addition, differences between farms indicates the influence of climatic and management condition of female recipients on pregnancy rate of embryos transferred.
